# How acceptable is adolescent self-consent for the HPV vaccination: Findings from a qualitative study in south-west England

**DOI:** 10.1016/j.vaccine.2020.09.074

**Published:** 2020-11-03

**Authors:** Suzanne Audrey, Michelle Farr, Marion Roderick, Karen Evans, Harriet Fisher

**Affiliations:** a Population Health Sciences, Bristol Medical School, University of Bristol, Bristol, UK; b The National Institute for Health Research Applied Research Collaboration West (NIHR ARC West) at University Hospitals Bristol NHS Foundation Trust, Bristol, UK; c Department of Paediatric Immunology & Infectious Diseases, Bristol Children’s Hospital, Bristol, UK; d Head of School Nursing (Sirona) and Specialist Nursing Services, Bristol and South Gloucestershire, UK

**Keywords:** HPV, Vaccination, Consent, Qualitative research, Adolescent health

## Abstract

**Background:**

Human Papillomavirus (HPV) vaccination programmes have the potential to reduce the incidence of cervical cancer. The preferred age for HPV vaccination is 12–13 years for optimal benefit. The legal framework in England allows adolescents to be vaccinated without parental consent if they are assessed as competent. A ‘South West Template Pathway on Self Consent for School Aged Immunisations’ was developed to improve uptake of immunisations in south-west England.

**Study aim:**

To examine how acceptable the new procedures are to the young women, parents and carers, school staff and immunisation nurses involved.

**Methods:**

The research was undertaken in two local authorities in south-west England during the 2017/18 and 2018/19 programme years. Semi-structured digitally recorded interviews were undertaken with 53 participants: one health service manager, three immunisation nurses, five staff at alternative education providers, three staff at mainstream schools, 19 young women and 22 parents. All recordings were transcribed verbatim and thematic analysis was undertaken, assisted by NVivo software.

**Results:**

Most participants were not fully aware of the legal framework that enables a young person to self-consent to vaccination. There was a strong presumption that parents should make decisions affecting the health of their children. The preferred age at which the HPV vaccination is administered (12–13 years) contributed to reluctance in endorsing self-consent which was thought to have the potential to break down trust between parents and school staff, and within families. In practice, formal self-consent was rare.

**Conclusion:**

Unresolved issues in relation to adolescent self-consent include public and professional perceptions of young people’s rights and abilities to take responsibility for decisions affecting their health, and concerns about the impact of self-consent on relationships both within families and between professionals and the families they serve.

## 1 Introduction

Human Papillomavirus (HPV) vaccination programmes for adolescent girls have the potential to reduce the incidence of cervical cancer [1]. However, lower socio-economic status, some ethnic groups, and being outside of ‘mainstream’ education are associated with lower uptake of HPV vaccination which may exacerbate existing inequities in the incidence of, and mortality from, cervical cancer [2], [3], [4].

For optimal benefit, the preferred age for HPV vaccination is before sexual debut, and the schools-based vaccination programme in England is predominantly administered in Year 8 when students are aged 12–13 years. In England the legal framework allows adolescents to be vaccinated without parental consent if they are assessed as Gillick competent (believed to have enough intelligence, competence and understanding to fully appreciate what is involved in their medical treatment) [5] but written parental consent is usually sought [6]. Immunisation nurses and school staff have shown reluctance to allow girls to self-consent to HPV vaccination [7], [8], [9] and a qualitative systematic review and evidence synthesis found, in a schools-based setting, the requirement for written parental consent presented the greatest barrier to accessing of the vaccine for some young women [10].

Public Health England (PHE) data for 2014/15 showed areas in the south-west of England with low uptake of HPV vaccination [11]. In response, staff at PHE South West developed new consent procedures which included student self-consent.

Before the new procedures were implemented, all female students in Year 8 (aged 12–13 years) were provided with an information leaflet about the HPV vaccination programme which they were asked to take home and discuss with their parents, together with a form asking parents or carers to provide written consent or refusal for their daughter to receive the vaccine. Only young women with a consent form signed by their parent, confirming permission for the vaccine, were invited to the school-based vaccination session.

Under the new procedures, information and consent forms were distributed as usual but included new information for parents indicating if a completed parental consent form was not returned the immunisation team would assess their daughter and invite her to self-consent if she could demonstrate understanding of the vaccination, in line the Gillick competency framework [5]. Consequently, young women without a signed parental consent form also attended the school vaccination session.

During the vaccination session, young women with a consent form signed by their parent received the vaccine. Those who wanted to receive the vaccine but had not returned a parental consent form spent more time with an immunisation nurse who attempted to contact a parent by telephone to ask for verbal parental consent. If this was given, the young woman received the vaccine. If a parent or carer could not be contacted, the immunisation nurse assessed the young woman’s competence, her understanding of the purpose of the vaccine and possible side effects, and any health issues that need to be taken into consideration. If she was deemed competent and indicated she had discussed the vaccine with her parents and it would not cause disagreement within the family if she was vaccinated, written consent was obtained and she received the vaccine. Young women who were not deemed competent, or indicated that vaccination would cause disagreement at home, were not vaccinated but given information about community-based clinics run by the immunisation nurses where the vaccine could be administered.

This paper focuses on qualitative research undertaken as part of a larger study [12] and considers how acceptable the new procedures were to young women, parents and carers, school staff and immunisation nurses.

## 2 Methods

The research was undertaken in two local authorities (LAs) in south-west England where uptake rates of the HPV vaccination programme were ranked 112th and 106th of 119 English LAs (excluding London). School recruitment took place during the 2017/18 and 2018/19 programme years. Mainstream schools in which at least 12 female Year 8 students were not vaccinated during the 2016/17 programme year were sent information about the study and were invited to participate. Of the 15 schools identified, four (26.7%) consented to take part. All alternative education provider settings (n = 17) were invited to participate in the study, of which five (29.4%) consented.

During the 2017/18 programme year, only four young women self-consented and all of them were given information about the study and invited to participate in an interview. However, written parental consent was required and this was not provided. Because of the relatively low number of young women self-consenting, in the 2018/19 programme year the inclusion criteria for young people’s interviews were expanded to include all Year 8 young women where a completed parental consent form for vaccination had not been received by the school. In addition, as the number of young women recruited in school settings was lower than anticipated, community groups for parents and young people in Bristol and South Gloucestershire were also approached (n = 18). Six community groups agreed to assist recruitment.

Topic guides were developed to cover the same key issues (beliefs about the HPV vaccine, views and experiences of the HPV vaccination programme, and opinions about the new consent procedures) with some adaptations relevant to the differing roles of immunisation nurses, mainstream school staff, alternative education providers, parents and young women. Digitally recorded interviews took place in schools, community organisations, private homes or by telephone, depending on the preferences of interviewees. Interviews were one-to-one, or in pairs or small groups, again to suit the participants.

All recordings were transcribed verbatim and thematic analysis was undertaken assisted by QSR NVivo software [13]. We used both an inductive and deductive approach to analyse the content, focusing on our main research questions while identifying key issues emerging from the data. Coding of all transcripts was undertaken by one researcher (HF), while a second researcher (MF) double-coded a sub-set of 12 transcripts to check for meaning, relevance and reliability. A series of consensus meetings (SA, HF, MF) were undertaken to review, refine and confirm the main themes and codes relevant to the acceptability of the new consent procedures. As the process of coding progressed and data were extracted, key terms and phrases were retained while repetition and extraneous text were removed (SA, HF, MF).

To illustrate key points, anonymised quotations have been chosen. The following identifiers have been allocated: A (alternative education provider), M (mainstream school), SS (school staff), CG (community group), P (parent), YW (young woman), IN (immunisation nurse), HM (health service manager); each followed by a number.

### 2.1 Funding and ethics

The study was funded by the National Institute for Health Research Research for Patient Benefit (NIHR RfPB) programme (project number PB-PG-0416-20013)**.** The University of Bristol Faculty of Health Sciences Research Ethics Committee and the National Health Service (NHS) Health Research Authority provided the required approvals. All interviewees gave written informed consent before participating in the study.

## 3 Results

Four mainstream schools (including one serving an inner-city ethnically diverse population, and another serving a predominantly White British low-income social housing estate) and five alternative education providers were recruited to the qualitative study. Semi-structured digitally recorded interviews were undertaken with 53 participants: one health service manager and three immunisation nurses who comprised the core immunisation team (all female); five school staff (four female, one male) at alternative education provision for young people with a range of physical and sensory disabilities, or with differing educational and behavioural needs; three staff at mainstream schools (two female, one male); 19 young women (eight Year 8 female students recruited through participating schools, and 11 young women aged 12–17 years attending community organisations), and; 22 parents (21 mothers and one father recruited through community organisations providing support for parents and families). Of the 19 young women interviewed: eight were from BAME communities; all of them received the HPV vaccine; 12 returned a signed parental consent form (one of whom had signed the form herself), six received the vaccine following parental verbal consent at the vaccination session, and 1 self-consented.

Although the new consent procedures included adolescent self-consent, it was rare in our study. Statistical analyses of the data across the two LAs, undertaken for the larger study, show only 0.3 percent of young women self-consented during the 2-year intervention period (Table 1). Parental verbal consent, community-based clinics and general practice surgeries were more likely routes to vaccination if written parental consent was not provided for the school vaccination sessions.

**Table 1:** Route to vaccination for young women who received the HPV vaccine.

School setting			Community catch-up clinics	General practice
*Parent written consent*	*Parent verbal consent*	*Adolescent self-consent*		
**N (%)**	**N (%)**	**N (%)**	**N (%)**	**N (%)**
5,538 (87.3)	299 (4.7)	17 (0.3)	217 (3.4)	219 (3.5)

The overarching themes presented below relate to understanding the legal framework, primacy of parental consent, vaccination beliefs, capacity to consent, prioritising relationships, and self-consent in practice.

### 3.1 Understanding the legal framework

If a young person in England is assessed to be competent, they have the right to accept or refuse health care including vaccinations. However, in our sample, parents were unclear about the legal framework and showed preference for delaying self-consent: “I think they have to be 16 to give consent for sexual activity so I think for injections and things like that, I still think it should be 16” (CG01P04).

School staff also appeared unsure of the current legal framework and legal implications of vaccinating without parental agreement: “We all think it’s the parents but actually they [*young women*] can give consent, is that correct?” (A04SS01); “My slight concern would be the, what would be the legal protection for the nurses doing the vaccines if they’d done that against the parents’ wishes” (A02SS01).

After the legal framework was explained during the interview, some young women began to argue self-consent was acceptable: “I think they should just stick to legally what’s right… I think there’s a lot more benefits that outweigh one angry parent emailing the school” (CG06YW02).

### 3.2 Primacy of parental consent

Almost all parents who were interviewed spoke about the importance of being part of the decision-making process, and some were particularly forthright: “As parents we make those decisions for our children on what we feel is best” (CG01P09); “You wanna stick a needle in my child without my permission, I’d knock you out” (CG01P01).

Young women also acknowledged the role of parents as the main consent providers: “It’s her parents whose, they’re in control of her” (CG05YW02); “I think you should still respect the parent because the parent is the one who has more knowledge in the situation” (M01YW02).

In contrast, some parents were less committed to the requirement for parental consent: “If they couldn’t get hold of me and she was in there, and they told me afterwards ‘We decided we didn’t have your consent, and we talked, and we felt she was competent and we went ahead’, I would not have a problem with that” (CG06P06); **“**I think the child should be allowed to have it done because I think it’s not the parents whose going be infected long term, it’s going to be the child” (CG02P02).

Similar views were expressed by young women: “I don’t think it’s fair if a child wants to have a vaccine for their future, so they don’t get ill, and their parents say no” (CG06YW03); “It’s her body so if she wants that, I think her parents should understand that if she wants to take the consequences, if they believe there are any, like it’s her decision” (CG06YW05).

School staff also articulated mixed views about the primacy of parental consent: “I think it should be down to the parents… I wouldn’t be happy for my children to be injected without my knowledge” (M01SS01); “I think as long as it’s all upfront and clear from the beginning then there’s absolutely no reason why a student can’t, shouldn’t self-consent” (M02SS01). Parental consent was perceived as particularly important in alternative educational settings: “I wouldn’t dream of giving a vaccination without [*parental*] consent I’m afraid, here, in this sort of setting” (A02SS01).

The importance of parental decision-making was not only seen in terms of young women receiving the vaccination against their parents’ wishes. A frequently raised concern was whether, in realising they could make the choice, young women would refuse the vaccine: “There would be a tiny bit of me that would be worried if I wasn’t in the loop that she just made some crazy decision with her mates not to go ahead with it” (CG06P06); “I do think you’d have to be careful they weren’t just not having it done because of the fear of an injection” (CG02P02).

This perspective was confirmed by young women themselves: “I don’t think we’re allowed to make the decision completely by ourselves, and I don’t think we should either because most kids will go ‘Oh, it’s a needle, I’ll go out of it, I won’t do it’ but it’s actually quite important” (CG03YW01); “Who would want to have a jab, because it would be painful, so I think it’s better for parents to consent for their children” (CG05YW01).

While generally supporting the vaccination programme, school staff acknowledged that young people had the right to refuse even if their parents had signed a consent form: “I personally feel that everyone should have it, but I think you can’t absolutely force it on someone” (M02SS01).

### 3.3 Vaccination beliefs

Almost all interview participants were supportive of vaccinations, including the HPV vaccine, to prevent ill health. Only one parent spoke about her opposition to vaccination: “It was on the internet because obviously I read up about everything. There’s two doctors who’ve been sacked from their jobs ‘cos they said the vaccine, the HPV, is actually giving girls cancer and stopping them from having children” (CG01P01).

Professionals and parents discussed how autism had been linked with the measles, mumps and rubella (MMR) vaccination. But almost all participants, including parents of children with autism, discounted the link: “I mean he’s [*adolescent son*] got ADHD, possible Asperger’s but as far as I’m concerned that wasn’t because of vaccines he had” (CG01P09). It was, however, recognised that other parents may have concerns: “I think there’s more fear in special needs schools because there is still the anecdotal evidence that vaccines, particularly the MMR, have caused the harm” (AO2SSO1).

Some parents and young women were unsure if adolescent self-consent was appropriate where parents were opposed to vaccinations: “The concern is where you’ve got families that maybe are very anti and wouldn’t consent and that’s going to cause all kinds of logistical problems” (CG02P04); “I would want my kids to be vaccinated, I would think it would be a positive thing. But then it’s not going to be so positive if it’s somebody that didn’t want them to be vaccinated” (CG01P08); “I don’t agree with not having vaccines but if she’s still a child I don’t think she should go against her parents’ wishes” (CG05YW01).

But it was also suggested that young women should be able to override their parents’ opinions: “I think being able to say ‘No, actually I want a vaccine’, I think it’s really important because it is their health and it is their body and it is them that it’s going to affect” (CG03P01). One young woman described her own experience of being vaccinated despite her parents’ anti-vaccination beliefs: “We got given like a big sheet and my mum didn’t want me to get that [*HPV vaccine]* or the meningitis I think, so I signed them myself and got it done anyway” (CG04YW01).

### 3.4 Capacity to consent

An important concern for parents was whether young women eligible for the HPV vaccination were emotionally mature enough to self-consent: “I wouldn’t trust [*daughter*] to make a decision ‘cos she can’t even go to the shop and get it right” (CG01P01); “We know what teenagers are like, they will just choose just depending on how they feel in the moment” (CG01P07).

In schools for young people with additional educational needs, staff were often keen to ensure parental consent: “I’d say here it would be a capacity issue, are they are able to understand what they’re consenting to, so we would want parents to consent” (A01SS01). However, some staff wanted parity with mainstream provision: “If children in mainstream are being told that they can consent, then our children, the majority of them, would have that capacity to consent. I think they should have every opportunity the same as any child attending a mainstream school gets” (A03SS01); “If they’ve got the cognitive ability to understand what a vaccine was, what’s actually going to happen to them, and the consequences of it, then yeah” (A05SS01).

Although 16 years or older had been suggested as a suitable age for self-consent to vaccination, some parents acknowledged that decision-making skills were not simply related to age: “Some 13-year olds can make really sensible decisions, and others you can’t trust them to go to the shop” (CG02P04); “They might not be able to communicate it verbally or as sophisticated as adults can do but they have a feeling for themselves and they have their dignity and they can say ‘Yes, that makes sense’ or ‘I like that’ or ‘No, I have a feeling that’s not good for me’ and they can do that at every age” (CG06P07).

The views of school staff varied in relation to the age at which young women should be able to self-consent: “I still think that it’s a little bit young, 13, to be self-consenting” (M01SS01); “Once they get to Year 8 [*aged 12*–*13 years*] many of them, are in a good position I would say to make an informed choice” (M02SS01). It was suggested school staff should contribute to the process of assessing competence: “I think that the school should have some input as to whether they feel, with a professional judgement, that a particular student is capable or in a position to be able to self-consent” (M02SS01).

Young women also expressed differing opinions about whether age was a good indicator of decision-making capacity: “I mean, there are different mindsets and people have different intelligence at the same age” (CG05YW01); “If people are well-informed like via an assembly or something, I think at 12 you’re like, most people are probably conscious enough to make that decision for themselves” (CG04YW01); “If you’re really young then I don’t think you should be able to give consent, but if you’re like over the age of like 13 maybe, then that would be quite a suitable age” (M09YW01).

The immunisation team expressed difficulty in giving a straightforward opinion about whether Year 8 is the right time to encourage self-consent: “Year 8 is a hard one. Some of them are still babies when they come and talk to you, they can’t even say the word sex or pregnant without getting all embarrassed. And some of them are really mature, really sensible, really know their own mind and can give consent, so it’s a really tricky age. I would love to say yes they should all be able to consent for their own health matters and be able to consent for them but truly some of them are not mature enough so it’s a real split at that age I think” (IN002).

School staff and students also suggested the capacity of young women to make informed decisions about vaccinations depended on the quality of information provided: “Lots of students would have said no because they probably need to look online to see what it was, and there’s lots of lies, or it makes them scared” (M09YW04); “It’s really important that students have been educated about what the vaccination is, why they’re having the vaccination, something to do with the science behind it because otherwise they’re not in a position I’d say to be able to make that informed choice” (M02SS01).

### 3.5 Prioritising relationships

Relationships within families and between parents, school staff and health professionals were often valued above the legal rights of young women, and concerns were raised that the self-consent process might “break down the trust between the school and the parent… I would be really upset if I didn’t know about it and it took place and I hadn’t been able to have a conversation, I’d be angry” (CG02P02); “You need to still contact the parents because … they could kick up a fuss” (CG02P06).

One school staff member suggested parents were more likely to return a signed form, indicating refusal, if they were strongly against vaccination: “As long as it’s all upfront and clear from the beginning then there’s absolutely no reason why a student can’t, shouldn’t self-consent… they [*parents*] would be more likely to return the form if they didn’t want to have it done with a note saying ‘Look, I don’t consent, OK’ as opposed to just not returning the form at all” (M02SS01).

But the importance of protecting home-school relationships was stressed by school staff in mainstream schools and alternative education providers: “School, parent, child, so there’s a 3-way triangle which everyone is involved - the self-consenting kind of cuts the parent out of that triangle so then you’ve just got school, child” (M10T01); “I suppose ultimately parental relationships are really important to us… I would hate to drive a wedge in between us and the family” (A04SS01).

Parents discussed potential negative impacts on relationships within families if young women were encouraged to decide about vaccination without parental consent: “The relationship between child and parent is really important… you don’t want to have a rift between the child and the parent open up” (CG01P07); “No child should be in fear of something terrible happening at home for any decision they make” (CG03P01). Consequently, although self-consent might be considered a positive step for some, it might also be an unfair burden: “The child should not be left alone with that decision and with being torn apart, between the school says one thing and the parent says another thing” (CG06P07).

The immunisation team raised similar concerns: “We want them to have it, if they really want to have it done, but we don’t want to be causing them harm within that family and breaking those family relationships” (HM001). And young women also acknowledged potential problems at home if decisions were not made in conjunction with parents: “It’s not right the kid should be prevented from being able to make that choice because of the parent, but at the same time, you do have to protect the kid and make sure they won’t get into trouble for making that decision” (CG04YW02); “You shouldn’t leave it so that the daughter has to tell her parents that she’s got the vaccination without their consent” (CG06YW05).

Although less common, some young women felt it was preferable not to inform parents: “I don’t think the parents necessarily even have to know… if a young person wants the vaccine enough, they can perfectly well lie” (CG06YW02).

One young woman explained why she did not inform her parents after signing the consent form herself: “I think they would have just been slightly annoyed that I’d gone behind their backs and done it because I think they’re pretty convinced in their idea that it will give me autism. Probably it would have just annoyed them” (CG04YW01). Another young woman suggested: “You have to ask the young person first whether they want to involve, like the parents to be involved. I think that’s probably more important than parents being involved 100 percent of the time” (CG04YW01).

### 3.6 Self-consent in practice

Seeking verbal consent by telephoning parents, as a stage before resorting to self-consent, was generally thought to be a useful addition to the consent process: “Initially when we talked about self-consent there were lots of concerns. Certainly, as a team, that’s why we suggested we do parental verbal consents as a step before going to self-consent, so I think we’re quite happy with that” (HM001); “As long as school always says at the beginning of the phone call ‘Nothing to worry about’ because otherwise you think your child has fallen down the stairs and broken both their legs or something. Other than that, I can’t see any problem with that at all” (CG06P02).

However, seeking telephone consent was not without criticism as it may require a quick judgement by a parent who felt unprepared: “If the parents, let’s say, have a religious problem and they haven’t formed an opinion as yet and they feel emotional about the topic and they don’t know where the position is and all of a sudden you have a professional calling say ‘Yes or no please’. Now that creates quite a bit of pressure” (CG06P07).

Where parents could not be contacted, the preference for parental involvement still dominated the ‘self-consent’ process: “If the child can’t tell us that mum or dad want me to have it done then we don’t do self-consent” (IN002); “The parents have to have said that they have seen the form, and they’ve had to have chatted to the child about them wanting to have the immunisation, for me to be happy to self-consent them” (IN001).

Concerns were raised about young women being responsible for informing their parents that they had received the vaccination if self-consent was progressed: “It’s just a bit unfair to put that on a kid, I think. And they may never have discussed it so I don’t think, they either wouldn’t know, or they’d just say the wrong thing and then get into trouble at home or something which would be a bit unfair” (CG01P03).

The immunisation team shared some of their experiences of self-consenting young women: “We have had some people that we’ve self-consented and the parents have come back and said ‘Thank you very much’, you know, ‘I haven’t been very organised today, things have been a bit mad, I really did want her to have it done so that’s great, thank you very much’” (HM001); “We have had a couple I know that have called in most upset that we’d taken self-consent” (IN002).

One notable example was described in which a young woman said her parents wanted her to have the vaccine but she had forgotten the parental consent form: “She was very aware of what the vaccination was about… we felt she was able to give her consent so we went ahead and gave the vaccination on the basis of self-consent and then had a huge complaint from parents because actually they’d signed the form to say no but the girl hadn’t handed that in… I think she was a very bright girl and knew how to get it without her parents’ consent ‘cos she knew her parents would say no… that girl won’t get her second dose next year ‘cos now we know the parents have adamantly said no, she’s not to receive it… I think that’s very sad” (IN003). In this situation the immunisation team had followed the procedures and were supported by their employer and Public Health England. No further action was taken by the parents. While this example shows the young woman was listened to and assessed, it also illustrates unresolved issues relating to adolescent self-consent.

As has already been noted, the right to self-consent includes the right to refuse vaccination. The immunisation team spoke about the process they would go through if a young woman, whose parent had signed the consent form indicating they wanted her to receive the vaccine, did not want to be vaccinated: “I would make sure she had all the information and probably… once they’re one-to-one with you, have all the information, calmed themselves down, they will have it done, but again I’m not there to force… ultimately the decision is hers and I would tell her that actually, although it’s ideal to have it younger, that actually if she changes her mind in two or three years’ time that she can contact her GP practice and get it done there” (IN002).

## 4 Discussion

### 4.1 Key findings

Most interview participants were not fully aware of the legal framework enabling young people to self-consent to vaccination if they are competent to do so. There was a strong presumption that parental consent should be sought.

The preferred age at which the HPV vaccination is administered, 12 to 13 years, contributed to reluctance in endorsing self-consent. The perceived variation in the maturity of Year 8 students made it difficult for respondents to give an overall opinion about whether it is the right time to encourage self-consent. This could be even more pronounced for young women with complex developmental and educational needs. The potential for new consent procedures to highlight the right to refuse vaccination was also a concern.

Almost all interview participants held favourable beliefs about vaccinations, but there was uncertainty about whether adolescent self-consent was appropriate where parents were opposed to vaccinations. Concerns were expressed that self-consent procedures could undermine trust between schools and parents. Furthermore, although self-consent might be considered a positive step for some, concerns were raised that it could put an unfair burden on young people and may have a negative impact on family relationships. In some cases, it was proposed that it might be preferable not to inform parents if their adolescent daughter had received the vaccination against their wishes.

To avoid some of the tensions associated with self-consent for the HPV vaccination, the immunisation team included two steps in the new procedures. Seeking verbal consent by telephoning parents was predominantly welcomed. Where parents could not be contacted, the assessment of competency included asking young women the reasons for the parental consent form not being returned and enabled the immunisation team to consider delaying vaccination if there were concerns about disagreement within the family. This appeared to act as a barrier to self-consent, although it was thought preferable to the possibility of young women being placed in a difficult position at home or damaging trust between parents and the school or health practitioners.

In practice, formal self-consent was rare. Where it occurred, it was said to have worked well in some cases and less well in others. The example that raised the most concern was of a young woman who had deliberately gone against her parents’ wishes to receive the vaccination. In contrast, another young woman explained that she had avoided conflict with her parents by signing the form herself and simply not informing them.

### 4.2 Strengths and limitations

The views and experiences of a change in consent procedures are presented from the perspectives of a range of stakeholders. However, recruitment of parents and young women for interview proved difficult through the school system, and we were not able to recruit sufficient Year 8 students and their parents, but we increased the number of interviewees by recruiting through community organisations. Most of the interviewees were female, and a male perspective is limited in our study. Almost all participants held pro-vaccination beliefs and we are unable to provide direct insights into how the new procedures are viewed by parents, school staff or young women with strong anti-vaccination beliefs. Self-consent was rare in our study and we can only provide limited understanding of cases where self-consent took place. However, we do share insights into the ways in which the new procedures were implemented and adapted to fit more comfortably with the prevailing view of parents’ rights to make decisions on behalf of their adolescent children.

### 4.3 Implications for research and practice

Our findings support a scoping review examining young people’s healthcare which found that children and young people are willing and able to make decisions about their healthcare services, but the rhetoric of the choice agenda is not realized [14]. It has been argued that strategies are needed to challenge prevailing views of children requiring protection, so that it becomes more acceptable for them to be involved in decisions about their healthcare [15]. Our research suggests self-consent was not a preferred option for our sample of stakeholders. Some important issues appear unresolved and require further exploration. What further measures are required to support young people who wish to self-consent? Should parents always be informed if their adolescent child self-consents to vaccination and, if so, by whom and how? Are there unmet information needs which, if addressed, could support understanding and acceptance of young people’s rights to self-consent for vaccinations? From September 2019, the HPV vaccination programme has been extended to young men [16] but the views of male stakeholders are underrepresented and there is scope for more research in that area. In response to some of these issues, co-production of information materials for young people is being undertaken and examined in a new research study [17].

## 5 Conclusion

This study has highlighted unresolved issues in relation to adolescent self-consent for the HPV vaccination and potentially other vaccines and healthcare initiatives for young people. These include public and professional perceptions of young people’s rights and abilities to take responsibility for decisions affecting their health, and concerns about the impact of self-consent on relationships within families and between professionals and the families they serve.

## Declaration of Competing Interest

The authors declare that they have no known competing financial interests or personal relationships that could have appeared to influence the work reported in this paper.
